# Semi-quantitative digital analysis of polymerase chain reaction-electrophoresis gel: Potential applications in low-income veterinary laboratories

**DOI:** 10.14202/vetworld.2016.935-939

**Published:** 2016-09-03

**Authors:** John F. Antiabong, Mafora G. Ngoepe, Adakole S. Abechi

**Affiliations:** 1 Department of Molecular Microbiology, School of Biological Sciences, Flinders University SA, Bedford Park, 5042, Australia; 2 Department of Applied Biotechnology, National Veterinary Research Institute, Nigeria; 3 Onderstepoort Biological Products, Onderstepoort, Pretoria, 0110, South Africa

**Keywords:** applications, digital image analysis, ImageJ software, polymerase chain reaction-electrophoresis, polymerase chain reaction amplicon, quantitative polymerase chain reaction

## Abstract

**Aim:**

The interpretation of conventional polymerase chain reaction (PCR) assay results is often limited to either positive or negative (non-detectable). The more robust quantitative PCR (qPCR) method is mostly reserved for quantitation studies and not a readily accessible technology in laboratories across developing nations. The aim of this study was to evaluate a semi-quantitative method for conventional PCR amplicons using digital image analysis of electrophoretic gel. The potential applications are also discussed.

**Materials and Methods:**

This study describes standard conditions for the digital image analysis of PCR amplicons using the freely available ImageJ software and confirmed using the qPCR assay.

**Results and Conclusion:**

Comparison of ImageJ analysis of PCR-electrophoresis gel and qPCR methods showed similar trends in the *Fusobacterium necrophorum* DNA concentration associated with healthy and periodontal disease infected wallabies (p≤0.03). Based on these empirical data, this study adds descriptive attributes (“more” or “less”) to the interpretation of conventional PCR results. The potential applications in low-income veterinary laboratories are suggested, and guidelines for the adoption of the method are also highlighted.

## Introduction

Conventional polymerase chain reaction (PCR) is a prerequisite tool in molecular biology research. However, the data obtained can only be interpreted as either “positive” (detectable) or “negative (non-detectable).” Conventional PCR is an endpoint assay whereby the amplified PCR products (amplicons) can only be detected at the end of electrophoresis using ethidium bromide (EtBr) and other nucleic acid dyes [1]. Quantitative PCR (qPCR) is a more sensitive assay [2]. However, qPCR is still not a standard test tool in most veterinary laboratories and most importantly in less developed nations where access to the real-time PCR technology is limited or non-existent.

Conventional PCR assay is a prerequisite tool in the molecular diagnosis and now available in most veterinary pathology laboratories. However, the data obtained can only be interpreted as either “positive” or “negative (non-detectable).” To enable resources-limited laboratories in making an empirical judgment as regards the relative amount of the amplified PCR amplicon, we reasoned that adding descriptive attributes (“more” or “less”) to the interpretation of traditional PCR results could be helpful.

Using a periodontal disease (PD) model, this study describes an agarose gel image analysis method for the semi-quantitation of PCR amplicons. The semi-quantitative measurement of PCR amplicons was achieved by digital analysis of PCR-electrophoresis gels using a freely available image analysis software-ImageJ [3]. It is noteworthy that this software has been successfully applied in quantitative immunoblot assays [4,5] but has not been widely employed in the quantitation of nucleic acids. The aim of this study was to evaluate a semi-quantitative method for conventional PCR amplicons using digital image analysis of electrophoretic gel. The potential applications and guidelines for the adoption of the method are discussed.

## Materials and Methods

### Ethical approval

Specimen collection was performed by an authorized veterinarian as required by the Animal Ethics Guidelines and approval of the School of Biological Sciences, Flinders University of South Australia and the Zoos-South Australia (Adelaide and Monarto zoos).

### PCR assay

**Figure 1:**
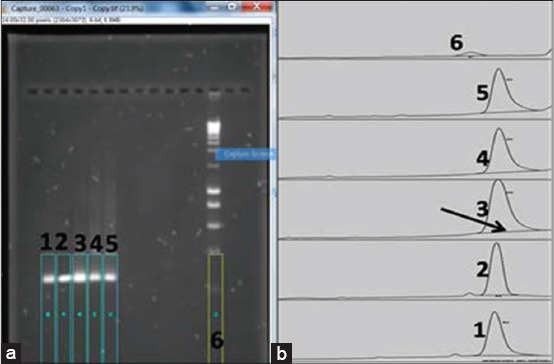
Screenshots of the ImageJ window showing the selected polymerase chain reaction (PCR) bands and molecular ladder (in rectangles). (a) Lanes 1-4=PCR positive bands (250 bp fragment of the *Fusobacterium necrophorum* encoded hemagglutinin-related-gene); Lane 5=Positive control; Lane 6=Molecular ladder (Hyperladder I) (Bioline; Australia). No amplification was observed in the negative control test sample. (b) ImageJ generated peaks of the corresponding PCR bands in Figure-1a. The peaks were based on the PCR band densities. The arrow indicates the line that specifies the “area” of measurement within the peak. The PCR band density is automatically generated by the ImageJ software using the specified “area.”

*Fusobacterium necrophorum* encoded hemagglutinin-related protein was amplified by PCR using 10 ng of the DNA extract from oral swabs of healthy and PD infected wallabies as described previously [6]. Electrophoresis of 5 μl of DNA molecular ladder (Hyperladder-I) (Bioline; Australia) and individual PCR amplicon was performed using a 2% agarose (Sigma; Australia) gel containing 0.5 μg/ml EtBr (Bio-rad; Australia) in ×0.5 tris-acetate-ethylenediaminetetraacetic acid (TAE) buffer (pH - 8) and run at 80 V for 60 min. The size, thickness of the agarose gel, reagents, and other conditions were kept constant. The TAE buffer was not reused to avoid any additive effect of residual EtBr on the PCR band density. The band-size and DNA concentration of each PCR amplicon was determined by comparison to the corresponding band in the molecular weight ladder (Hyperladder-I) (Figure-1a). The DNA concentration in each band of Hyperladder-I is predetermined by the manufacturer.

The amplicon images (PCR bands) in the gel were captured under ultraviolet (UV) light and documented using a Digi Doc gel documentation system (Bio-rad; Australia). All the parameters and experimental conditions used were kept constant throughout the study. The image was saved (in tiff. format) on a computer for digital image analysis using ImageJ software version 1.4.3u.

### Digital analysis of PCR-electrophoresis gel

The PCR bands were analyzed as follows (the arrows (→) and symbols [^#^ or *] indicate specific mouse-click steps for the image analysis on the ImageJ window):

On the ImageJ window, click on file → Open → Select the gel image from the computer file location → Image → Type → 8-bit → (Optional step: Click on “invert image” to have the “Peak plot” in an upright format) → Process → Filters → Gaussian blur (an optional step to straighten graphs with too many peak slopes thereby making it easy to apply the “Straight and Wand” tools later in the image analysis process) →^#^ “Rectangular” tool → Select bands to be compared on the gel image by drawing a rectangle to cover specific bands of interest (Figure-1a) → Analyse → Gels →^ϒ^ First lane (this creates a rectangle on the band). Note: Repeat step→^#^ “Rectangular” tool, to step →^ϒ^ First lane, for the second band of interest or left click on the first rectangle and move it to the second band of interest. →^β^ Next lane. (Note: Repeat step →^#^ “Rectangular” tool, to step→^β^ Next lane, for every other band of interest. → Analyse → Gel → Plot lanes (the “Straight” tool on the ImageJ window becomes selected automatically) → draw a line from one end of the curve to the other to select the peak. If minor peaks (“spikes or clefts”) are observed ensure that the line starts from the right bottom-end of the minor peak to the right bottom-end of the major peak of interest (Figure-1b). → Wand → click on the inside of each peak in succession (e.g. bands 1 to 4 according to how the bands were selected with the “Rectangular” tool above) → Analyse → Gel → Label peaks (this will compute the relative percentage of the peaks of each band and the result will appear on a pop-up window) → File (on the pop-up window) → Save As → Save.

To save the graph (Peak plot), click on the graph and then select File on the main window of ImageJ software → Save. To document the gel image used for a specific analysis, right-click on the image and select “capture screen.” The processed image screen is captured and can be saved on the computer (Figure-1a and b). The ImageJ software is downloadable at rsbweb.nih.gov/ij/.

Hyperladder I (Bioline; Australia) was used for the determination of the band size and estimation of the DNA concentration in each PCR band by comparing the band density of a band in the Hyperladder I to a PCR band of interest (Figure-1a). The DNA concentration in each band of Hyperladder I was predetermined by the manufacturer. The agarose gel image analysis method was compared to qPCR data.

### qPCR analysis

The qPCR on the *F. necrophorum* encoded hemagglutinin-related protein was performed and analyzed as described previously using 10 ng of DNA extract from the same set of samples [6]. The result was presented as a percentage of the template DNA (10 ng).

### Statistical analysis

The Wilcoxon rank sum test method was used to determine the level of significant difference at p≤0.05.

## Results and Discussions

ImageJ analysis of PCR-electrophoresis gel and qPCR assays of the same DNA extracts revealed a similar trend in the amount of amplified DNA in the tested samples (Table-1 and Supplemental Figures-S1 and S2) and showed that there were more *F. necrophorum* DNA in the PD cases (ImageJ analysis=26.7 ng/μl; qPCR=8.9%) (p≤0.03) as compared to the healthy cases (ImageJ analysis=6.2 ng/μl; qPCR=0.003%) (p≤0.03). This was in concordance with the findings in a previous study [6]. Although qPCR is more sensitive than an end point assay, Schmittgen *et al*. [7] the method reported in this study can provide an affordable means of getting an insight into the possible trend that may exist between specimen types/conditions investigated. This allows a preliminary determination of the choice of the specimen with the potential to always yield a reliable result for routine PCR in resources-limited laboratory. As molecular testing is expected to complement other forms of veterinary diagnosis [8], it is important to strive for accuracy even in under-funded laboratories.

**Table-1:** Semi-quantitation of *F. necrophorum* DNA in healthy and PD oral swab specimen: Comparison between qPCR and endpoint PCR-electrophoresis gel estimation (ImageJ analysis) of PCR bands (amplicons) densities.

ImageJ analysis (DNA qPCR (%)* concentration (ng/μl)			

PD	Healthy	Periodontal disease	Healthy
35.9	21.7	13.4	0.0052
25.35	13.9	10.5	0.0065
23.15	0	0.643	0
22.61	0	0.421	0
26.7	8.9	6.2	0.003
Wilcoxon rank sum test	0.03	Wilcoxon rank sum test	0.03

* The qPCR-estimated concentration was expressed as a percentage of the template DNA concentration (10 ng of DNA extract from oral swab specimen). *F. necrophorum*: *Fusobacterium necrophorum*, PD: Periodontal disease, qPCR: Quantitative polymerase chain reaction

**Figure-S1:**
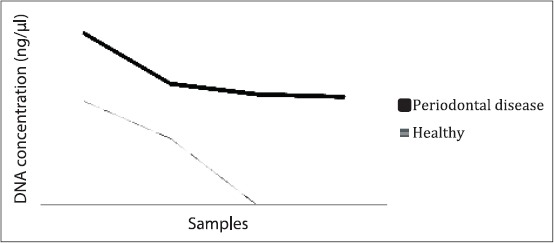
A three-dimensional image showing the trend of *Fusobacterium necrophorum* DNA concentration in healthy and periodontal disease (PD) samples obtained from captive wallabies. ImageJ analysis of endpoint polymerase chain reaction (PCR)-electrophoresis gel was used to estimate the density of the PCR bands (amplicons) which was then compared to a band of known DNA concentration. *F. necrophorum* DNA concentration was higher in PD (mean=27 ng/μl) than in healthy participants (mean=8.9 ng/μl). The samples were run in duplicates and equal volume from each replicate was pooled, and 5 μl was loaded onto the agarose gel.

**Figure-S2:**
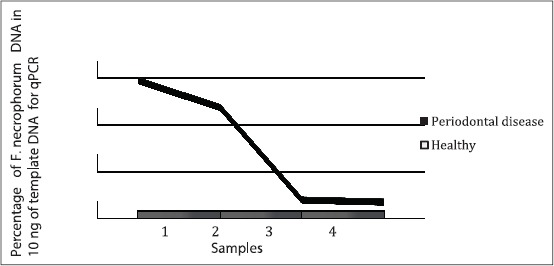
A three-dimensional image showing the trend of *Fusobacterium necrophorum* DNA concentration in healthy and periodontal disease (PD) samples obtained from captive wallabies. *F. necrophorum* DNA was higher in PD (mean=6.24%) than in healthy participants (mean=0.0029%). The quantitative polymerase chain reaction estimated concentration was expressed as a percentage of the template DNA concentration (10 ng) of DNA extract from oral swab sample). The samples were run in duplicates.

Moreover, based on empirical data, this method adds descriptive attributes (“more” or “less”) to the interpretation of traditional PCR results and could be applied especially in resources-limited nations where access to the real-time PCR technology is limited or non-existent. Potential applications includes: (i) Preliminary investigations that seek to compare conditions such as “treated and non-treated” or “diseased and healthy” to determine the trend (“more” or “less”) of the target gene of interest and (ii) making a decision on the choice of pathological specimen needed for an accurate PCR diagnostic regime. This is of particular importance as veterinary pathogens are known to show preferences to particular host tissue(s)/organ(s) [1], often referred to as the predilection sites.

**Figure 2:**
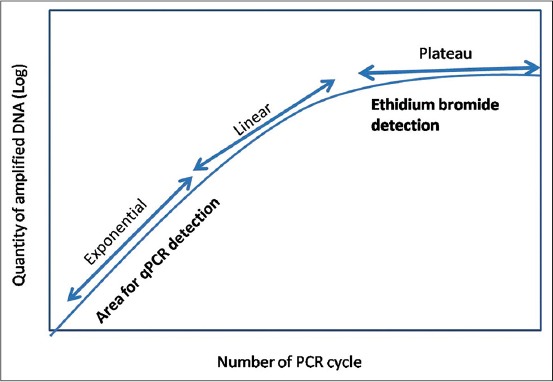
Polymerase chain reaction (PCR) phases in log view showing the region of ethidium bromide detection of PCR amplicons. The quantitative PCR method is detected at the exponential phase.

To apply the method described in this report, in-house optimization is recommended to factor-in laboratory specific conditions. The following points should be noted at the experimental design stage: (A) ImageJ software measures the individual band densities by relative comparison of all the band characteristics in a single gel [3]. Therefore, to ensure accuracy, it is important to run all samples on the same gel. The trends observed in different gel analyses can then be compared, (B) the size, thickness, density, picture format of the agarose gel, reagents, and other conditions should be kept constant. The limitations of the described semi-quantitative method are acknowledged and hence to further improve the reliability/utility of the method the following should be noted:

- Constant UV exposure time can be achieved using image analysis hardware with adjustable exposure time. Agarose gel imaging equipment with such capability is now commercially available
- PCR amplification reaches a plateau due to enzyme and/or substrate limitation and the exertion of inhibitory effect on the enzymes by the accumulated PCR product [9,10]. At this plateau, the differences between test samples may be obscured, and it becomes difficult to accurately quantify the initial DNA concentrations in different test samples in an endpoint PCR [11]. This may affect the utility of gel image analysis for DNA quantitation and calls for further optimization of the method to improve accuracy. The proposed improvement to the method is discussed below:
  - Determination of the limit of detection of ImageJ quantitation of PCR amplicons.
  - On the basis of the DNA concentration at the upper and lower limits of ImageJ quantitation of PCR amplicons, perform a qPCR to determine the cycle number before the beginning of the plateau phase (i.e., the area within the linear phase region). This is the region where EtBr detection is possible (Pfaffl, 2004) (Figure-2).
  - The determined cycle number for the lower limit of ImageJ quantitation should be taken as the cut-off cycle number for ImageJ quantitation of PCR amplicons.

It should be noted that in spite of this optimization, the ImageJ quantitation of PCR amplicons will still be semi-quantitative because the quantitation is not performed at the exponential phase of the PCR amplification reaction. The exponential phase yields more accurate quantitation in standard qPCR method (Figure-2) [11].

## Conclusion

Finally, the method described in this study is not intended to replace the qPCR method but rather, to serve as a preliminary indicator of the possible trend that may exist across specimen/conditions evaluated to make quick empirical decisions on the choice of specimen for PCR assay. This will be especially useful in a new disease outbreak situation (e.g. emerging pathogen); in low-income countries with under-funded veterinary pathology laboratories, pending when such laboratories are able to establish collaboration with laboratories abroad for qPCR for confirmation of their results - a process that often takes a while based on our practical experiences. This technique may also be useful in the preliminary definition of the potential trend in the gene copy number of a pathogen in an ecological niche using appropriate PCR primers, as demonstrated in this study.

## Authors’ Contributions

Concept and design: JFA. Data acquisition and analysis: JFA, MGN, and ASA. Drafting and revision of the manuscript: JFA, MGN, and ASA. All authors read and approved the final manuscript.
